# A Mechanistic Assessment of the Discordance between Normal Serum Alanine Aminotransferase Levels and Altered Liver Histology in Chronic Hepatitis B

**DOI:** 10.1371/journal.pone.0134532

**Published:** 2015-07-31

**Authors:** Xianqiong Gong, Jiaen Yang, Jinmo Tang, Chong Gu, Lijian Huang, Ying Zheng, Huiqing Liang, Min Wang, Chuncheng Wu, Yue Chen, Manying Zhang, Zhijian Yu, Qianguo Mao

**Affiliations:** 1 Hepatology Unit, Xiamen Hospital of Traditional Chinese Medicine, Xiamen, Fujian, China; 2 Shenzhen Key Lab for Endogenous Infection, Shenzhen Nanshan Hospital, Shenzhen, Guangdong, China; Singapore Institute for Clinical Sciences, SINGAPORE

## Abstract

To understand the mechanisms underlying the discordance between normal serum alanine aminotransferase (ALT) levels and significant alterations in liver histology of chronic hepatitis B virus (HBV) infection with persistent normal ALT (PNALT) or minimally elevated ALT. A total of 300 treatment-naive chronic HBV-infected patients with PNALT (ALT ≤ upper limit of normal [ULN, 40 U/ml]) or minimally elevated ALT (1-2×ULN) were retrospectively enrolled. All patients underwent liver biopsy and histological changes were analyzed along with biochemical and HBV markers. Among 300 participants, 177 were HBeAg-positive and 123 HBeAg-negative. Significant histologic abnormalities were found in 42.9% (76/177) and 52.8% (65/123) of HBeAg-positive and HBeAg-negative patients, respectively. Significant fibrosis, which is a marker of prior injury, was more frequently detected than significant necroinflammation (suggesting active liver injury) in both HBeAg-positive and -negative groups, suggesting that liver injury occurred intermittently in our cohort. No significant differences were noticed in the percentage of patients with severe fibrosis between HBeAg-positive and negative phases or between ages 30 and 40 and over 40, suggesting that the fibrosis was possibly carried over from an early phase. Finally, lowering ALT ULN (30 U/L for men, 19 U/L for women) alone was not adequate to increase the sensitivity of ALT detection of liver injury. However, the study was limited to a small sample size of 13 HBeAg-positive patients with ALT in the revised normal range. We detected significant liver pathology in almost 50% of chronic HBV infected patients with PNALT (ALT ≤ 40 U/ml) or minimally elevated ALT. We postulated that small-scale intermittent liver injury was possibly responsible for the discordance between normal serum ALT and significant liver changes in our cohort.

## Introduction

Chronic hepatitis B virus (HBV) infection represents a significant public health burden. Approximately 400 million people are affected by chronic HBV infection worldwide. Chronic HBV-related liver injury constitutes a major risk factor for development of end-stage liver disease including cirrhosis and hepatocellular carcinoma in HBV endemic region[1–3].

Liver injury and associated liver pathology in chronic hepatitis B can be mitigated by current antiviral treatment, as evidenced by normalization of elevated serum alanine aminotransferase (ALT) and improvement of liver histology in treated patients. Since the main objective of current antiviral therapy was to block the progression of chronic liver injury, a significant portion of chronic HBV-infected patients who had persistent normal ALT (PNALT) or elevated ALT less than 2 times of the upper limit of normal (ULN), were not suitable for antiviral treatment[4–6], because ALT levels in those patients suggest absence or minimal liver injury[7,8]. However, increasing evidence points to a disturbing picture in which about 20–50% of patients with normal ALT exhibited significant necroinflammation and fibrosis in liver biopsy, and such patients face a high risk of cirrhosis and other end stage clinical complications without treating liver injury[9–12]. Importantly, the fundamental mechanisms associated with normal or nearly normal ALT despite liver injury, remain unknown. Furthermore, the chronic HBV-infected patients with normal or minimal elevated ALT consist of two subpopulations based on HBV replication status: HBeAg-positive patients with high serum HBV DNA level and the HBeAg-negative group with lower HBV DNA level. Significant changes in liver histology were also observed in patients with normal ALT, but with HBeAg-negative and lower HBV DNA level. It is important to determine if the main altered liver histology is carried over from the previous liver injury or occurred in the later phase of HBV infection. Different scenarios require different treatment strategies.

As stated above, the traditional ALT ULN value (40 U/L) to determine the eligibility of patients for treatment initiation may miss those patients who are experiencing significant changes in hepatic histopathology without detectable elevation in ALT. Investigators have suggested lowering the ULN of ALT[13–15] to address current ALT deficiency in reflecting liver injury. A study of 6835 Italian healthy subjects by Prati et al, suggested that the ULN of ALT should be adjusted to 30 U/L for men and 19 U/L for women[16]. However, some studies doubted were if a lower ULN of ALT improved the effectiveness of management of chronic HBV-infected patients [12,17].

In this study, we present our analysis of liver histology in a large cohort of Chinese chronic hepatitis B (CHB) patients with PNALT or minimally elevated ALT. Our analysis was focused on investigating mechanisms underlying normal or nearly normal ALT under conditions of liver injury and fibrosis. We determined if the primary liver histopathology in later HBeAg-positive phase or HBeAg-negative patients resulted from early liver injury or recent episodes, and evaluated the sensitivity of lowering ALT ULN in detecting liver injury. We propose that a repeated, intermittent small-scale liver injury along with chronic infection in our cohort contributed to normal or nearly normal ALT levels. However, such small-scale liver injury despite intermittent features can cause severe consequences in the long term.

## Patients and Methods

### Patients

We evaluated all CHB patients who were admitted to the Hepatology Unit of Xiamen Hospital of Traditional Chinese Medicine from January 1, 2010 to October 1, 2014. A total of 300 treatment-naive CHB patients who underwent percutaneous liver biopsy as part of clinical evaluation, were included in this study. The inclusion criteria were as follows: All patients were hepatitis B surface antigen (HBsAg)-positive for at least 12 months, with HBV DNA levels greater 500 IU/ml, and PNALT or minimally elevated ALT. PNALT was defined by continually normal ALT levels tested at least on 3 occasions over a 1- year period prior to liver biopsy, whereas minimally elevated ALT levels were defined as ALT levels ranging between ULN and 2×ULN[18]. Patients with the following conditions were excluded from the study: (1) evidence of concomitant etiologies including chronic hepatitis C or D coinfection or superinfection, autoimmune hepatitis, alcoholic liver disease, non-alcoholic fatty liver disease, Wilson’s disease, and drug-induced liver injury; or (2) HIV coinfection or evidence of immune suppression.

The study protocol was approved by the ethics committee of Xiamen Hospital of Traditional Chinese Medicine. Written informed consent was obtained from all subjects.

### Liver biopsy and histology

All patients underwent percutaneous liver biopsy guided by ultrasonography. Liver biopsies were obtained using 16G biopsy needles. A qualified biopsy specimen was either a minimum 1.5 cm long or displayed 6 or more portal tracts. Two serial sections were stained with hematoxylin-eosin-safran and Masson’s trichrome, respectively. Scheuer’s scoring system was used to semi-quantify the histologic necroinflammation from G0 to G4 and fibrosis stages from S0 to S4 by the same pathologist, who was blinded to the biochemical and virologic results of the patients. Significant histological abnormality was defined as necroinflammation grade ≥ G2 and/or fibrosis stage ≥ F2.

### Laboratory tests

Serum samples were collected within 3 days prior to liver biopsy. Serum biochemistry tests including albumin (ALB), globulin (GLB), ALT, aspartate aminotransferase (AST), a-fetoprotein (AFP), prothrombin time (PT) and complete blood cell counts were determined by commercial kits. The ULN of ALT and AST were set at 40 IU/L for both male and female. HBV serological markers were detected using electrochemiluminescence immunoassay (Roche Diagnostics, Germany). HBV DNA was quantitatively determined by a HBV DNA qPCR kit with a low detection limit of 500 IU/mL (Amplly Bioteh.Co, Ltd, China).

### Statistical analysis

All statistical analyses were performed using SPSS software package version 22.0. Continuous variables were presented as mean ± standard deviation (or median and range) and Mann-Whitney U test was used for comparison of non-parametric continuous variables. Categorical variables were expressed as frequency and percentage and analyzed by Chi-square test. Multivariate logistic regression was used to determine the independent predictors of significant histologic abnormalities. A two-sided P value < 0.05 was considered as statistically significant.

## Results

The baseline characteristics of our cohort are listed in Table 1. Of the 300 enrolled patients, 177 (59.0%) were HBeAg-positive and the remaining 123 (41.0%) were HBeAg-negative. In HBeAg-positive group, 50 (28.2%) and 127 (71.8%) patients were categorized as PNALT and minimally elevated ALT, respectively, while 51 (41.5%) and 72 (58.5%) were categorized into PNALT and ALT 1–2×ULN, respectively, in the HBeAg-negative group.

**Table 1:** Baseline characteristics of 300 chronic hepatitisB patients.

Patient Characteristic	All Patients(n = 300)	HBeAg-positive (n = 177)			HBeAg–negative (n = 123)		
		PNALT(ALT≤40 U/L)	Minimally elevated ALT(ALT > 40 U/L)	P-value	PNALT(ALT≤40 U/L)	Minimally elevated ALT(ALT > 40 U/L)	P-value
**Mean age**	36(28–43)	30(25–41)	30(26–39)	NS	41.76±9.55	42.94±9.72	NS
**Male**	218(72.67%)	29(58%)	88(69.29%)	NS	41(80.39%)	60(83.33%)	NS
**ALB (g/L)**	43(40–45)	43(40–45)	42(40–45)	NS	43.20±3.45	42.57±3.53	NS
**GLB (g/L)**	33(30–36)	32(31–36)	34(30–36)	NS	31.86±3.56	33.18±4.36	NS
**ALT(U/L)**	51(35–66)	32(26–37)	62(52–71)	< 0.001	26.90±7.34	57(51–69)	<0.001
**AST(U/L)**	51(35–66)	29(23–35)	41(34–52)	< 0.001	25(22–28)	40(33–52)	<0.001
**WBC (×10** ^**9**^ **/L)**	5.40(4.60–6.30)	5.48±1.34	5.60(4.80–6.60)	NS	5.30(4.70–6.20)	5.30(4.23–6.20)	NS
**PLT (×10** ^**9**^ **/L)**	175.07±54.24	189.46±65.62	178.39±48.53	NS	167.45±54.59	164.61±53.11	NS
**PT (s)**	13.00(12.50–13.60)	12.75(12.40–13.20)	13.00(12.50–13.80)	0.036	13.00(12.50–13.50)	13.05(12.40–13.50)	NS
**AFP(ng/mL)**	2.88(2.04–4.26)	2.44(1.87–3.38)	2.30(3.26–4.50)	0.007	2.34(1.86–3.08)	3.46(2.40–5.62)	< 0.001
**HBV DNA (log IU/mL)**	6.12(4.52–7.25)	7.10(5.55–7.95)	7.14(6.17–8.11)	NS	4.18(3.25–4.44)	5.03±1.05	< 0.001

Continuous variables are expressed as mean ±SD or median and interquartile range, and categorical variables are described by count and proportions. Abbreviations: PNALT, persistent normal ALT; ALB, albumin; GLB, globulin, ALT, alanine aminotransferase; AST, aspartate aminotransferase; WBC, white blood cells; PLT, platelet; PT, prothrombin time;AFP, α-fetoprotein; HBV, hepatitis B virus; NS, not significant.

**Fig 1:**
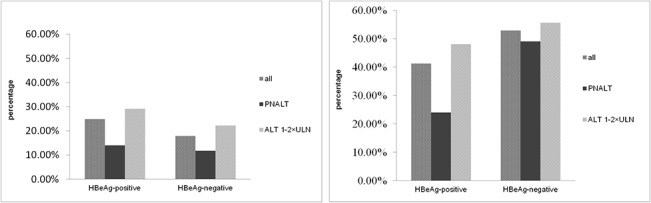
Distribution of significant necroinflammation and fibrosis among 300 chronic hepatitis B patients. (A) Significant necroinflammation (≥ G2) was found in 24.9%, 14.0% and 29.1% in all patients, PNALT (ALT ≤ 40 U/ml) and ALT 1–2×ULN subgroups in HBeAg-positive patients, and 17.9%, 11.8% and 22.2% in all patients, PNALT and ALT 1–2×ULN subgroups in HBeAg-negative patients, respectively. (B) Significant fibrosis (≥ S2) was found in 41.2%, 24.0% and 48.0% in all, PNALT and ALT 1–2×ULN subgroups in HBeAg-positive patients, and 52.9%, 49.0% and 55.6% in all, PNALT and ALT 1–2×ULN subgroups in HBeAg-negative patients, respectively. Frequencies of significant necroinflammation and fibrosis in HBeAg-positive patients with ALT 1–2×ULN were much higher than in patients with normal ALT (P < 0.05).

**Fig 2:**
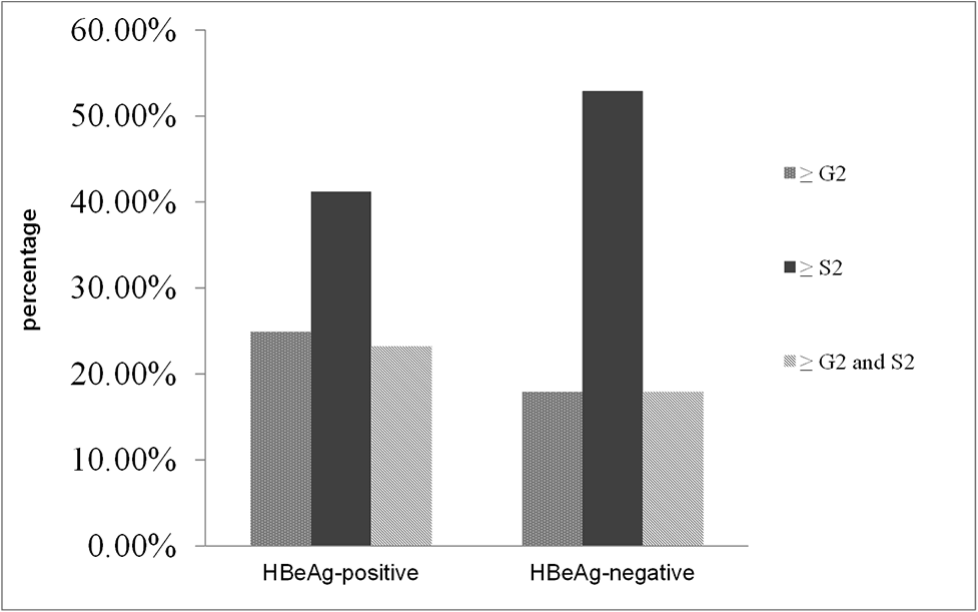
Incidence of necroinflammation ≥ 2, or fibrosis ≥ 2 or a combination of both ≥ G2 and S2. Significant levels of necroinflammation (≥ G2), fibrosis (≥ S2), and necroinflammation (≥ G2) and fibrosis (≥ S2) occurred in 24.9%, 41.2%, and 23.2%, respectively, in HBeAg-positive patients. Significant levels of necroinflammation (≥ G2), fibrosis (≥ S2), and necroinflammation (≥ G2) and fibrosis (≥ S2) were seen in 17.9%, 52.9%, and 17.9%, respectively, of the HBeAg-negative patients.

The percentages of liver necroinflammation and fibrosis in all groups are shown in Fig 1. Nearly 42.9% (76/177) in HBeAg-positive group and 52.8% (65/123) in HBeAg-negative group exhibited significant changes in liver pathology. Among the 177 HBeAg-positive patients, 14.0% (7/50) in patients with PNALT and 29.1% (37/127) in ALT 1–2×ULN subgroup showed significant necroinflammation, while 24.0% (12/50) and 48.0% (61/127) showed significant fibrosis, respectively. The fibrosis detected was more frequent than necroinflammation in the HBeAg-positive group (P = 0.001). The percentage of patients who carried both ≥ G2 and S2 changes was smaller than the patients with ≥ S2 change alone (Fig 2), suggesting that the resolved liver injury (see as fibrosis) was more frequent than the active liver injury at biopsy. Frequencies of significant necroinflammation and fibrosis in patients with ALT 1–2×ULN were much higher than in patients with normal ALT (P < 0.05).

In HBeAg-negative group, necroinflammation was detected in 11.8% (6/51) in patients with normal ALT, 22.2% (16/72) in subgroup with ALT 1–2×ULN, while significant fibrosis was found in 49.0%(25/51) and 55.6% (40/72) of these patients, respectively. No significant difference in liver pathology was found between subgroups of HBeAg-negative patients. However, fibrosis was more frequently detected than necroinflammation in HBeAg-negative group. The percentage of patients who carried both ≥ G2 and S2 changes was smaller than those with ≥ S2 change alone (Fig 2), suggesting that the liver injury reflected by the necroinflamationary scores, was intermittent during the course of chronic HBV infection in the majority of our cohort. There was no significant difference in the percentages of patients with significant fibrosis in both HBeAg positive and negative groups, suggesting that fibrosis was possibly carried over from early phase.

The normal clinical values of ULN of ALT suggested by Prati et al (i.e. 30 U/L for men and 19 U/L for women) were analyzed in patients with normal ALT. There were 13 HBeAg-positive patients with ALT levels within the normal range. No patient had significant necroinflammation or fibrosis. However, 32.4% (12/37) of patients with ALT levels greater than the normal range showed significant fibrosis (P < 0.05). No significant difference in both necroinflammation and fibrosis was found between the two subgroups of HBeAg-negative patients (Table 2). Our results suggest limited utility of lowering the ULN of ALT.

**Table 2:** Characteristics of CHB patients with PNALT analyzed using revised ULN (ALT of 30 U/L for men and 19 U/L for women).

Patient Characteristic	HBeAg-positive			HBeAg-negative		
	ALT ≤ revised ULN	ALT > revised ULN	P-value	ALT ≤ revised ULN	ALT > revised ULN	P-value
**Mean age**	32.54±10.10	29 (25.5–40.5)	NS	39 (34.5–44.0)	42.17±8.75	NS
**Male**	10 (76.92%)	19 (51.35%)	NS	24 (85.71%)	17 (73.91%)	NS
**ALB**	42.39±5.42	43 (40–44)	NS	43.61±3.21	42.70±3.73	NS
**GLB**	34.00±2.89	33.19±4.60	NS	31.57±3.77	31 (30–35)	NS
**ALT**	23.39±5.20	35 (31–38)	< 0.001	22.14±5.54	33 (31–37)	< 0.001
**AST**	23.54±3.45	31 (26.5–37.5)	< 0.001	22.50±3.16	28 (26–31)	< 0.001
**WBC (×10** ^**9**^ **/L)**	6.08±1.25	5.28±1.33	NS	5.25 (4.75–6.30)	5.30±1.06	NS
**PLT (×10** ^**9**^ **/L)**	186.46±48.45	190.51±71.23	NS	174.57±54.07	158.78±55.16	NS
**PT**	12.79±0.84	12.80 (12.40–13.20)	NS	12.75 (12.43–13.38)	13.11±0.85	NS
**AFP**	1.91±0.85	2.63 (1.93–4.00)	0.009	2.34 (1.87–2.96)	2.18 (1.69–3.14)	NS
**HBVDNA(log IU/mL)**	7.99±0.54	6.20 (5.03–7.45)	< 0.001	3.45 (3.20–4.29)	4.44±0.85	0.006
**necroinflammation grade**						
**< G2**	13 (100%)	30 (81.08%)	NS	26 (92.86%)	19 (82.61%)	NS
**≥ G2**	0 (0%)	7 (18.92%)		2 (7.14%)	4 (17.39%)	
**Fibrosis stage**			<0.05			NS
**< S2**	13 (100%)	25 (67.57%)		16 (57.14%)	10 (43.48%)	
**≥ S2**	0 (0%)	12 (32.43%)		12(42.86%)	13 (56.52%)	

**Fig 3:**
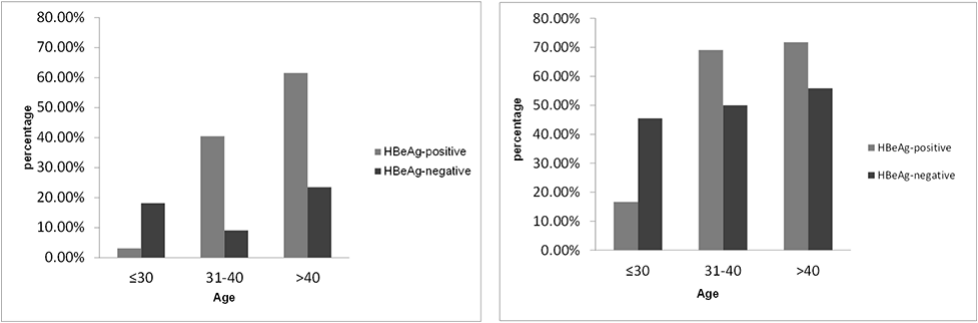
Distribution of significant hepatic histopathology among different age subgroups. (A) Significant necroinflammation (≥ G2) occurred in 3.1% of subgroups of patients ≤ 30 years, 40.5% in 31–40 years and 61.5% in > 40 years in HBeAg-positive patients, respectively; and in 18.2% of subgroups of patients ≤ 30 years, 9.1% in 31–40 years and 23.5% in > 40 years in HBeAg-negative patients, respectively. (B) Significant fibrosis (≥ S2) was found in 16.7%, 69.1% and 71.8% of HBeAg-positive patients in the age subgroups of ≤ 30, 31–40 and >40, respectively, of and in 45.5%, 50.0% and 55.9% of HBeAg-negative patients in the age categories of ≤ 30, 31–40 and > 40, respectively. Significant differences in necroinflammation (P < 0.001) and fibrosis (P < 0.001) occurred among subgroups of HBeAg-positive patients. HBeAg-positive patients over 30 years showed much higher percentages of significant histologic abnormalities than those ≤ 30 years of age (P < 0.001).

Patients were further stratified by age. The distribution of significant hepatic histopathology among different age subgroups is shown in Fig 3. Significant differences in necroinflammation (P < 0.001) and fibrosis (P < 0.001) were found among subgroups of HBeAg-positive patients. Further, significant histologic abnormalities were much higher in HBeAg-positive patients aged above 30 years than those ≤ 30 years of age (P < 0.001). However, no significant difference was found between the age group of 31–40 and those over 40. There was no significant difference in necroinflammation and fibrosis among different age subgroups of HBeAg-negative patients.

Clinical parameters that were analyzed by the multivariate regression correlated positively with liver histological abnormalities (Table 3). Age (P < 0.001), AST (P < 0.001) and AFP (P = 0.002) were independently associated with significant necroinflammation and age (P < 0.001), AST (P < 0.001), HBV-DNA (P = 0.007) and PLT (P = 0.002) were independently associated with significant fibrosis in HBeAg-positive patients. In HBeAg-negative group, AST (P = 0.016) and ALB (P = 0.006) independently correlated with significant necroinflammation, and male gender (P = 0.021), PT (P = 0.001), ALB (P = 0.002) and GLB (P = 0.017) independently correlated with significant fibrosis.

**Table 3:** Multivariate analysis of clinical parameters independently associated with significant histological abnormalities.

Patient Characteristic		Clinical parameter	OR	(95%CI)	P-value
**HBeAg-positive**	necroinflammation grade ≥ G2	age	5.186	2.740–9.814	< 0.001
		AST	1.061	1.028–1.095	< 0.001
		AFP	1.553	1.180–2.045	0.002
	fibrosis stage ≥ S2	age	2.918	1.759–4.839	< 0.001
		AST	1.073	1.039–1.108	< 0.001
		HBV-DNA	0.658	0.486–0.892	0.007
		PLT	0.985	0.977–0.995	0.002
**HBeAg-negative**	necroinflammation grade ≥ G2	AST	1.048	1.009–1.088	0.016
		ALB	0.805	0.689–0.941	0.006
	fibrosis stage ≥ S2	male	4.173	1.242–14.029	0.021
		PT	2.509	1.424–4.423	0.001
		ALB	0.784	0.674–0.913	0.002
		GLB	1.158	1.026–1.307	0.017

Abbreviations: AST, aspartate aminotransferase; AFP, α-fetoprotein; HBV, hepatitis B virus; PLT, platelet; ALB, albumin; GLB, globulin, PT, prothrombin time; OR, odds ratio; CI, confidence interval.

## Discussion

The focus of this study was to unravel the mechanisms underlying the normal or nearly normal ALT values despite histologic evidence suggesting liver injury in a significant number of chronic HBV-infected patients. We analyzed the liver pathology in 300 treatment-naive patients who were PNALT or ALT 1–2×ULN along with HBV and biochemical markers. We found that 24.0% vs 48.0% of HBeAg-positive, and 49.0% vs 55.6% of HBeAg negative patients with PNALT and minimally elevated ALT, respectively, carried significant changes in liver pathology. The percentages of abnormal liver histology in our cohort were within the ranges reported by other studies. For instance, a study from Hong Kong showed that 22.5% HBeAg-positive patients with normal ALT had significant histologic abnormalities[19]. Another study found that 37% of patients with PNALT had significant fibrosis or inflammation [11]. In a Chinese cohort, significant fibrosis occurred in about 70% of patients (HBeAg positive or negative) who had minimally elevated ALT [12], suggesting that the previous notion of chronic HBV-infected patients with PNALT had “healthy” livers may be inaccurate.

Importantly, fibrosis was significant and more frequent than necroinflammation in both groups at the time of liver biopsy. Our understanding of this finding is that liver injury reflected by necroinflammation in our cohort, or broadly in chronic HBV-infected patients with normal ALT or nearly normal ALT was intermittent, but not constant, implying that the earlier injury was resolved or repaired before advent of new injury. We anticipate similar or higher frequencies of necroinflammation compared with fibrosis, which follows liver injury if there was constant necroinflammation. However, the fibrosis was much higher, suggesting that persistent or news liver injury in the same patients with significant fibrosis (a marker of previous liver injury) was less frequent. Our findings are consistent with another Chinese cohort study [12]. Thus the liver injury may occur at a tempo of damage and repair, then the same process may repeat after an interval. This intermittent injury is different from persistent liver injury that presumably generates persistently elevated ALT as often seen in a portion of chronic HBV-infected patients, and is underappreciated in the field. In addition to the intermittent features, the scale of liver injury is also a factor determining if ALT levels would be sufficiently elevated for detection. Serum ALT level is largely determined by the amount of enzyme released from the damaged hepatocytes. Thus the degree of liver damage is directly related to the ALT levels in the serum. Therefore, patients with minimally elevated ALT showed significantly higher percentages of prominent liver injury than PNALT (29.1% vs 14.0% in the HBeAg-positive patients and 22.2% vs 11.8% in the HBeAg-negative patients). Our observation was similar to the previous reports [19,20]. We can infer that the scale of liver injury in our cohort was smaller than in patients with ALT > 2×ULN. We further suggest that the scale of liver injury in patients with PNALT was smaller than in patients with 1–2× ULN ALT. Thus, we propose that a minor liver injury that occurred intermittently in our cohort was largely responsible for PNALT or nearly normal ALT. However, such intermittent, small-scale liver injury carries significantly higher risk for progression to end-stage liver disease over years or decades. Accumulation of such small-scale liver injuries is often ignored by physicians and patients, raising the question of clinical management of the condition. It does not seem to be sustainable with current no treatment approach to those patients. In this study no significant difference in significant necroinflammation and fibrosis was found between the age group of 31–40 and over 40 years in HBeAg- positive patients, or among different age subgroups of HBeAg-negative patients. Since more than 90% of chronic HBV infections in China result from perinatal infection, the patient’s age can be used to infer approximately, the years of chronic HBV infection. Our results show no further significantly increased percentage of necroinflammation or fibrosis even when patients are extending course of chronic HBV infection, or arrive in later HBeAg phase or HBeAg-negative phase. It appears that the percentage of detectable necroinflammation remains relative steady in both early and later phases of chronic HBV infection, suggesting that intermittent liver injury occurs at a similar pace regardless of the phases of chronic infection in our cohort. However, the significant fibrosis found in the later HBeAg phase or in the HBeAg-negative phase was possibly carried over from earlier phase though the severity of fibrosis may be increased in the later phase. Our results imply that fibrosis starts in the HBeAg phase in nearly 50% patients with PNALT or 1–2×ALT. To mitigate the severity of fibrosis in those patients, therapeutic interventions including antiviral treatment in the earlier phase of HBV infection should be a priority, for better outcome.

We also investigated the clinical utility of lower ALT normal ranges suggested by Prati. In HBeAg-positive group, a higher frequency of significant fibrosis was found in patients with ALT levels exceeding Prati’s normal ranges than those within the ranges. However, we only had 13 patients with ALT within the revised normal range. A large cohort is needed to validate this finding. Furthermore, there was no difference in liver pathology in HBeAg-negative group when re-categorizing 51 patients with the revised ranges. One study suggests reducing the threshold ULN of ALT in HBeAg-positive patients with normal ALT [21]. Our results suggest a limited value of lowering the ALT ULN to 30 U/L for men, or 19 U/L for women. The real challenge is not only the need to lower the ULN, but also to increase the frequency of ALT testing since the liver injury most probably occurred intermittently in our cohort. Infrequent testing of ALT may not be the best strategy to detect transiently elevated ALT.

One of the implications of our results is that HBeAg-positive patients with high HBV DNA and normal ALT levels frequently manifested significant histological abnormalities in our cohort and other studies [12] although they were initially considered immunotolerant. In fact, the characterization of patients with immune tolerance has already been questioned. Increasing evidence suggests that clonal hepatocyte repopulation, an indirect measure of targeted destruction of HBV-infected hepatocytes, occurs in patients in the immunotolerant phase [22–24]. Further investigations are needed in such patients. The second implication is that patients with minimally elevated ALT showed significantly higher percentages of prominent liver injury and fibrosis than PNALT. They should be very carefully managed, especially those with a family history of HCC or cirrhosis. Liver biopsy or non-invasive fibrosis assessment are strongly recommended. Thus, those patients should be the next group of eligible patients for antiviral treatment, if the current recommendation is expanded. The third implication is related to more than half of HBeAg-positive patients who were over 30 years old with PNLALT or minimally elevated ALT who manifested a higher frequency of significant fibrosis. A liver biopsy should be considered for such patients.

There are several limitations in this study. This is a retrospective cross-sectional study without any follow-up data after biopsy. In addition, patients’ HBV genotypes were not investigated since HBV genotyping is not routinely performed in clinical practice.

In summary, we detected significantly altered liver histology in almost 50% of chronic hepatitis B patients with persistent normal or minimally elevated ALT. We further elucidated the mechanisms of discordance between the appearance of normal or nearly normal ALT in the serum and the significant changes in liver pathology. We propose that small-scale liver injury, occurring intermittently is possibly responsible for such discordance. The fibrosis detected in the late HBeAg or in HBeAg-negative phase was possibly carried over from an early HBeAg phase, supporting therapeutic intervention in early HBeAg positive patients, as a priority. Lowering ALT ULN and increasing the frequency of ALT testing are recommended for management of patients with transiently elevated ALT.
